# Different types of agricultural land use drive distinct soil bacterial communities

**DOI:** 10.1038/s41598-020-74193-8

**Published:** 2020-10-15

**Authors:** Shin Ae Lee, Jeong Myeong Kim, Yiseul Kim, Jae-Ho Joa, Seong-Soo Kang, Jae-Hyung Ahn, Mincheol Kim, Jaekyeong Song, Hang-Yeon Weon

**Affiliations:** 1 Agricultural Microbiology Division, National Institute of Agricultural Sciences, Rural Development Administration, Wanju, Republic of Korea; 2 Water Supply and Sewerage Research Division, National Institute of Environmental Research, Incheon, Republic of Korea; 3 Research Institute of Climate Change and Agriculture, National Institute of Horticultural and Herbal Science, Rural Development Administration, Jeju, Republic of Korea; 4 Soil and Fertilization Division, National Institute of Agricultural Sciences, Rural Development Administration, Wanju, Republic of Korea; 5 Korea Polar Research Institute, Incheon, Republic of Korea

**Keywords:** Microbial communities, Biogeography, Microbial ecology

## Abstract

Biogeographic patterns in soil bacterial communities and their responses to environmental variables are well established, yet little is known about how different types of agricultural land use affect bacterial communities at large spatial scales. We report the variation in bacterial community structures in greenhouse, orchard, paddy, and upland soils collected from 853 sites across the Republic of Korea using 16S rRNA gene pyrosequencing analysis. Bacterial diversities and community structures were significantly differentiated by agricultural land-use types. Paddy soils, which are intentionally flooded for several months during rice cultivation, had the highest bacterial richness and diversity, with low community variation. Soil chemical properties were dependent on agricultural management practices and correlated with variation in bacterial communities in different types of agricultural land use, while the effects of spatial components were little. *Firmicutes*, *Chloroflexi*, and *Acidobacteria* were enriched in greenhouse, paddy, and orchard soils, respectively. Members of these bacterial phyla are indicator taxa that are relatively abundant in specific agricultural land-use types. A relatively large number of taxa were associated with the microbial network of paddy soils with multiple modules, while the microbial network of orchard and upland soils had fewer taxa with close mutual interactions. These results suggest that anthropogenic agricultural management can create soil disturbances that determine bacterial community structures, specific bacterial taxa, and their relationships with soil chemical parameters. These quantitative changes can be used as potential biological indicators for monitoring the impact of agricultural management on the soil environment.

## Introduction

Diverse soil microbes play critical roles in plant growth and health. They decompose organic compounds and participate in the recycling of nutrients, such as nitrogen, phosphorus, and potassium, which are important for plant growth^1–3^. Some soil microbes in the rhizosphere and endosphere of plants improve tolerance to abiotic and biotic stress^4^. In addition to physicochemical properties of soils, soil microbial communities largely determine agricultural productivity^5^. To develop sustainable agriculture, understanding of ecological features of microbiomes in agroecosystems is needed.

The biogeography of soil microbial communities has been investigated at various spatial scales. Fierer and Jackson (2006) observed that microbial biogeography is primarily controlled by edaphic variables, not geographic distance. Another study of microbial communities in soils collected across the state of California, USA, showed that land-use types such as coastal grasslands, inland grasslands, deserts, coniferous forests, freshwater wetlands, and perennial and annual agricultural fields were closely associated with distinct microbial communities at a regional level^6^. A more recent and detailed characterization of soil microbial communities reported different biogeographic patterns of soil microbial communities across natural forests with vegetation gradients and distinct edaphic variables^7^. Different patterns of microbial diversity across different habitats (e.g., alpine grassland, desert, desert grassland, and typical grassland) were also observed in the drylands of northern China^8^. These investigations together suggest that the types of habitats or land use affect biogeographic patterns of bacterial taxa from regional to continental scales.

Agricultural management such as fertilization, irrigation, and tillage are important factors that affect the biodiversity and function of terrestrial ecosystems and can also lead to soil ecosystem degradation^9–13^. Previous studies show that land management practices such as chemical fertilization have a significant effect on bacterial community structure^14–16^. Effects of soil parameters, including pH, electrical conductivity (EC), carbon and nitrogen contents, salinity, and texture, on microbial community composition have been reported in many studies^17–22^, and this relationship was shown to be significant even in unique environments, such as the black soils of Northeast China^23–25^.

Bacterial taxa with distinct relative abundance patterns have been proposed as potential biological indicators that reflect environmental conditions. A recent study by Hermans et al. showed that microbial communities across diverse New Zealand soil types (e.g., indigenous forest, exotic forest, horticulture, and dairy) are more sensitive to changing soil environments than to variation in climate or increased geographical heterogeneity^26^. They also observed certain dominant taxa to be significantly related to specific soil parameters. These results support the use of specific bacterial taxa and their relative abundances as biological indicators that can be used to predict various soil attributes (e.g., pH, nutrient concentrations).

To explore interactions between microbial taxa in complex soil microbial ecology, co-occurrence network analysis has been widely used^27,28^. In the network, keystone taxa that have frequent interactions with many others are predicted to play an important role in microbial ecology^29^. Distinct co-occurrence patterns have been reported in different agricultural practices (organic and conventional farming)^16^ and habitats (bulk soil and rhizosphere)^30^. However, the co-occurrence networks of soil bacterial communities in different types of agricultural land use have not been explored using a large number of samples.

To elucidate the soil microbial distributions in agricultural soils, we collected 853 soil samples from four major types of agricultural land, including greenhouses, orchards, paddy fields, and uplands, throughout the Republic of Korea. We measured the edaphic factors of the soils and performed 16S rRNA gene pyrosequencing analysis of bacterial communities. The specific objectives of this study were to characterize bacterial communities in different agricultural land-use types through analyses of bacterial community diversity, composition, indicator species, and co-occurrence patterns.

## Results

### Bacterial community variation across different agricultural land-use types

To survey bacterial communities in agricultural soils across the Republic of Korea, we collected 853 soil samples from four major types of agricultural land use: greenhouses (211), uplands (209), orchards (224), and paddy fields (209) (Supplementary Fig. S1 and Supplementary Data S1). We obtained a total of 3,616,347 high-quality sequence reads by 16S rRNA gene pyrosequencing and identified 68,528 operational taxonomic units (OTUs) based on 97% sequence identity across all samples.

**Figure 1:**
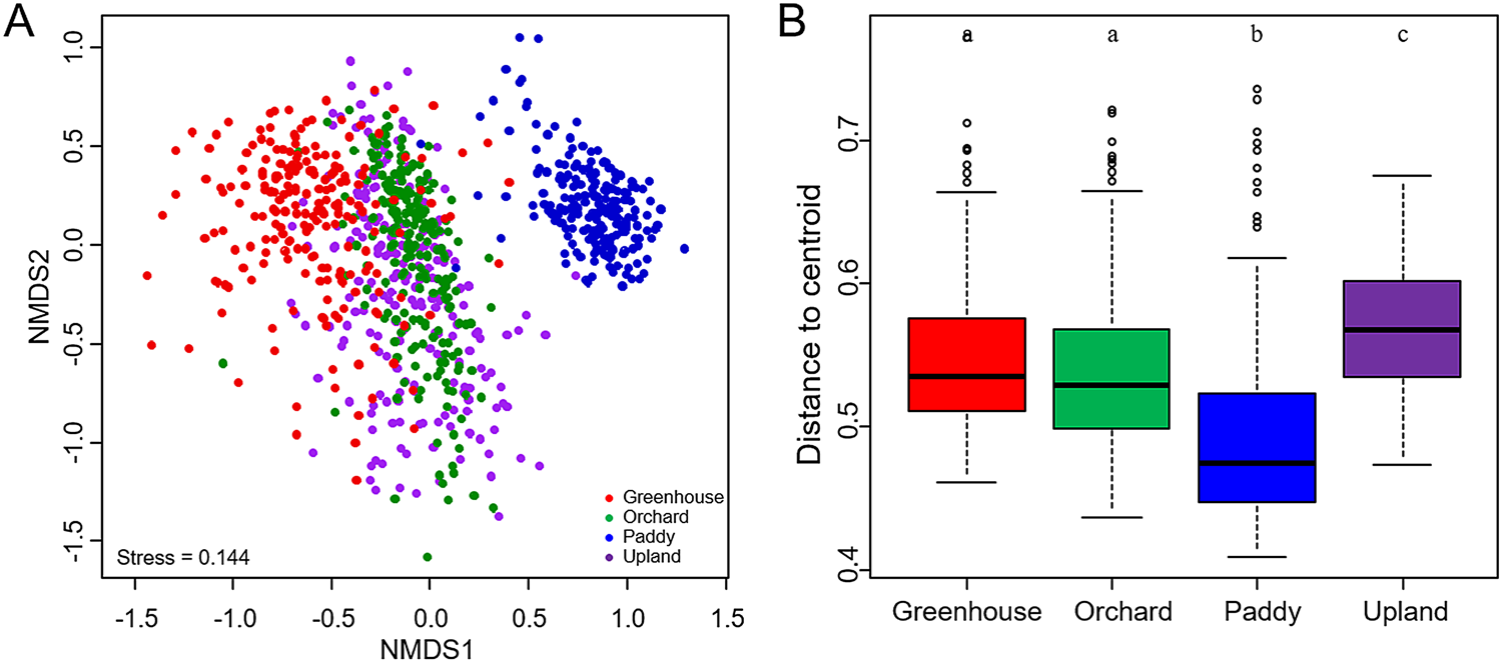
Beta-diversity of soil bacterial communities in the four types of agricultural land use. Non-metric multidimensional scaling (NMDS) ordination of soil bacterial communities (**A**). Box plot illustrating the beta-dispersion of bacterial communities (**B**). Significant differences between land-use types were tested by Tukey’s HSD and are indicated by different letters (P < 0.05). Boxes represent the interquartile range (IQR), and whiskers indicate the furthest point within 1.5 × IQR above or below the IQR. Values beyond this range are plotted as individual points. The central line indicates the median.

The variation in bacterial community structures was visualized with a nonmetric multidimensional scale (NMDS) plot based on Bray–Curtis distance. Bacterial communities in paddy soils were clearly differentiated from those in the other types of soil (Fig. 1A). Bacterial communities in greenhouse soils were also differentiated from those in orchard and upland soils. The significant differences between agricultural land-use types were confirmed by pair-wise comparison analysis of similarities (ANOSIM) and permutational multivariate analysis of variance (PERMANOVA) (Supplementary Table S1). Although bacterial communities of orchard and upland soils were closely positioned in the ordination plots (Fig. 1A), the pair-wise comparison tests were significant for all pairs of agricultural land-use types (Supplementary Table S1). The dispersion of soil bacterial communities within each type of agricultural land use was examined by measuring the distance between the centroid. Bacterial community dissimilarity within each type of agricultural land use was the lowest in paddy soils and the highest in upland soils (Fig. 1B).

To compare alpha-diversity between the samples, the OTU dataset was sub-sampled to the smallest number of total reads within a sample (1,002 reads). Chao1 and ACE richness estimators were significantly higher in paddy soils, while those in upland soils were lower (Table 1). Similarly, paddy soils showed significantly higher Shannon and inverse-Simpson diversity indices, followed by orchard, greenhouse, and upland soils. Taken together, paddy soils had significantly higher bacterial richness and diversity, with lower bacterial community variation, while upland soils harbored bacterial communities with lower richness and diversity but greater variation compared to other types of agricultural land use. Although the compositions of bacterial communities in upland and orchard soils look similar according to NMDS, the greater bacterial community variation in upland soils may partially explain the significant difference from orchard soils.

**Table 1:** Alpha-diversity for soil bacterial communities in the four different types of land use.

Land-use type	No. of OTUs	Coverage	Richness estimator		Diversity index	
			Chao-1	ACE	Shannon	Inverse-Simpson
Greenhouse (n = 211)	564 ± 80	0.60 ± 0.07	1529 ± 353^b^	2566 ± 692^b^	5.9 ± 0.3^c^	340 ± 183^c^
Orchard (n = 224)	581 ± 85	0.59 ± 0.08	1506 ± 366^b^	2374 ± 713^c^	6.0 ± 0.3^b^	403 ± 200^b^
Paddy (n = 209)	623 ± 49	0.54 ± 0.05	1778 ± 319^a^	3107 ± 855^a^	6.1 ± 0.2^a^	447 ± 157^a^
Upland (n = 209)	514 ± 80	0.66 ± 0.07	1172 ± 300^c^	1764 ± 585^d^	5.8 ± 0.3^d^	290 ± 147^d^

The original dataset was sub-sampled to 1,002 reads.

^a–c^The letters in each column indicate significant differences (P < 0.05, Tukey’s HSD).

*OTUs* operational taxonomic units.

### Variation in soil chemical properties across different types of agricultural land use

**Figure 2:**
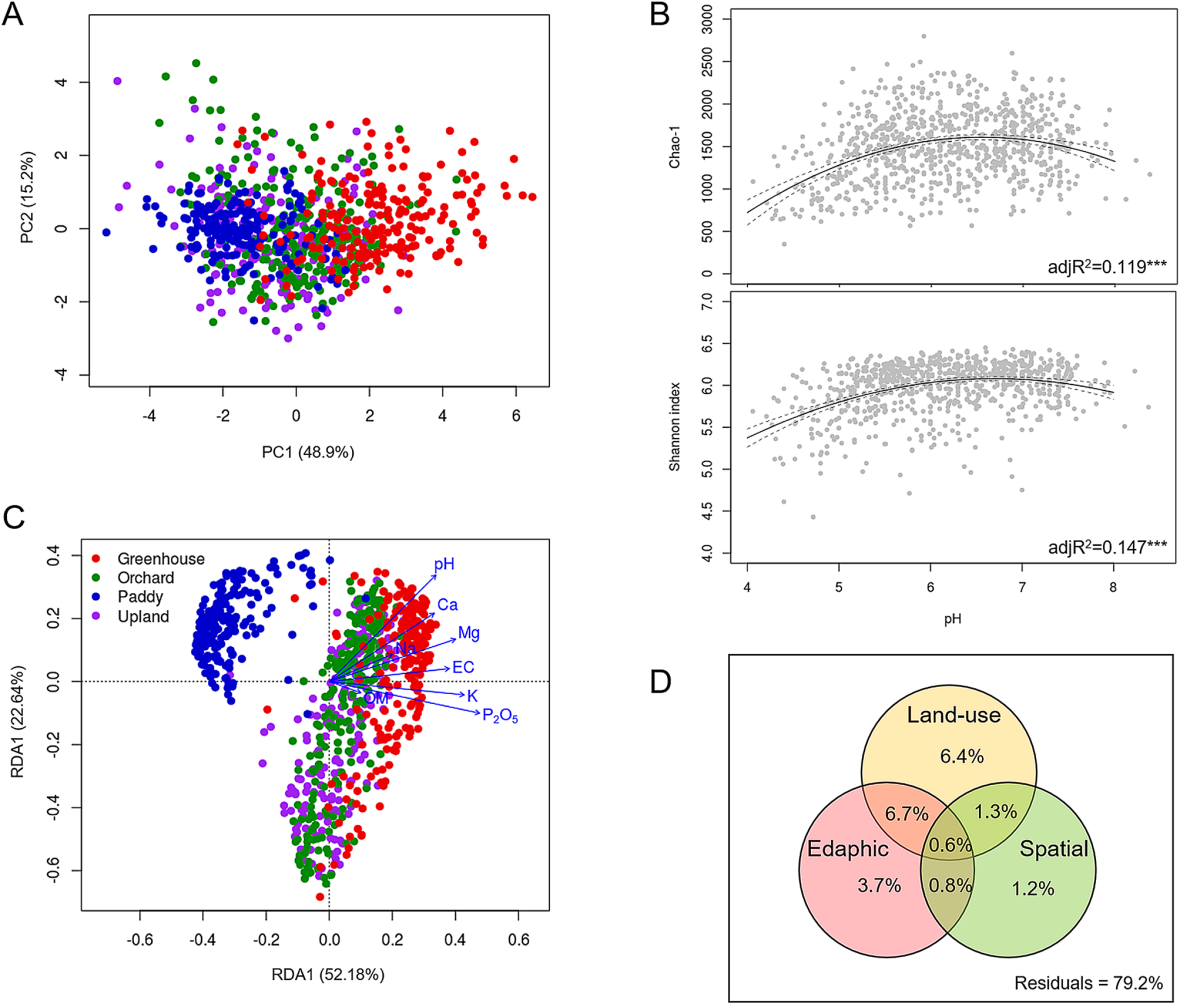
Soil chemical properties associated with the types of agricultural land use. Principal component analysis (PCA) of soil chemical properties using z-transformed soil variables (**A**). The association of bacterial richness (Chao-1) and diversity (Shannon index) with soil pH in different types of agricultural soils (**B**). Quadratic regression was used to determine the adjusted R^2^ values and statistical significances (***P < 0.001). Redundancy analysis (RDA) of bacterial communities constrained by soil chemical properties (**C**). The joint biplot indicates the correlation between the chemical factors and ordination scores of RDA axes. *EC* electrical conductivity, *OM* organic matter. Venn diagram representing variation partitioning of bacterial communities explained by land-use types, edaphic and spatial variables (**D**).

The principal component analysis (PCA) ordination plot showed that soil chemical properties were clearly separated between paddy and greenhouse soils along the first axis, which explains 48.9% of total variation, and those in upland and orchard soils were in between (Fig. 2A). Of the soil properties we measured, EC, available P_2_O_5_, and exchangeable cations (Ca^2+^, Mg^2+^, K^+^, and Na^+^) were significantly higher in greenhouse soils compared to soils of other types of agricultural land use, while paddy soils had significantly lower values of pH and available P_2_O_5_, Mg^2+^, and K^+^ (Table 2).

**Table 2:** Description of soil chemical properties in the four different types of land use.

Land-use type	Statistics	pH (1:5)	EC (dS m^−1^)	OM (g kg^−1^)	Av. P_2_O_5_ (mg kg^−1^)	Ex. cation (cmol_c_ kg^-1^)			
						K^+^	Ca^2+^	Mg^2+^	Na^+^
Greenhouse (n = 211)	Mean ± S.D	6.5 ± 0.8^a^	3.9 ± 3.9^a^	41.0 ± 22.6^a^	1,023 ± 576^a^	1.7 ± 1.3^a^	11.4 ± 4.7^a^	3.7 ± 1.9^a^	0.9 ± 2.1^a^
	Range	4.3–7.8	0–21.0	8.1–184.8	57–3,018	0.1–8.5	0.9–28.7	0.4–10.0	0.1–29.1
Orchard (n = 224)	Mean ± S.D	6.2 ± 0.9^a^	0.7 ± 0.8^b^	38.6 ± 29.1^a^	670 ± 391^b^	0.9 ± 0.7^b^	7.5 ± 3.7^b^	2.0 ± 1.1^b^	0.2 ± 0.2^b^
	Range	4.2–7.9	0.1–7.4	2.7–178.9	24–1,911	0.1–5.1	0.5–19.4	0.2–6.2	0.0–0.7
Paddy (n = 209)	Mean ± S.D	5.8 ± 0.6^b^	0.5 ± 0.4^b^	30.1 ± 25.5^b^	139 ± 156^c^	0.3 ± 0.2^c^	5.7 ± 2.7^c^	1.3 ± 0.9^c^	0.3 ± 0.3^b^
	Range	4.6–7.5	0.1–2.5	6.0–165.0	6.3–1,098	0.1–1.0	0.8–20.1	0.2–4.5	0–2.0
Upland (n = 209)	Mean ± S.D	6.1 ± 0.9^a^	0.6 ± 0.6^b^	25.5 ± 23.5^b^	589 ± 428^b^	0.8 ± 0.6^b^	6.1 ± 3.7^c^	1.7 ± 0.9^b^	0.2 ± 0.2^b^
	Range	4.1–8.4	0.1–4.1	4.2–236.0	21–1,789	0.1–3.4	0.3–29.8	0.1–5.6	0.0–0.8

^a–c^The letters in each column indicate significant differences (P < 0.05, Tukey’s HSD).

*EC* electrical conductivity, *OM* organic matter, *CV* coefficient of variation.

Among the edaphic factors measured, bacterial richness (Chao-1) and diversity (Shannon index) had a significant association with soil pH (Fig. 2B). Bacterial richness and diversity were the highest in neutral soils and lower in acidic soil, which is consistent with previous studies that utilized a variety of biogeographical scales and land uses^6,17,23,31,32^. In particular, bacterial richness and diversity in orchard soils showed the strongest correlation with soil pH, while those in paddy soils with lower pH levels (pH 5.0–6.0) showed no significant correlation (Supplementary Fig. S2 and S3).

The redundancy analysis (RDA) ordination plot constrained by soil chemical properties also showed that bacterial communities were separated by agricultural land-use types along the first axis (Fig. 2C). The chemical properties we measured in this study explained 14.1% of the total variation. The triplots show that EC and K^+^ are important factors in the dispersion of the bacterial communities along the first axis. We identified specific OTUs that are highly correlated with chemical properties (Pearson r > 0.5, *P* < 0.01). Only six OTUs were correlated with EC, which belonged to the families *Rhodospirillaceae* (OTU4130, OTU5485, OTU340, and OTU505) and *Rhodobacteraceae* (OTU4767) and the phylum *Chloroflexi* (OTU269) (Supplementary Fig. S4).

Although many studies have reported that microbial community similarity tends to decrease along increasing geographical distances^33,34^, no significant distance-decay patterns of bacterial communities in agricultural soils were observed (Supplementary Fig. S5). Variation partitioning analysis was performed with three explanatory components—land-use types, edaphic and spatial variables. The spatial variables were generated via Moran’s eigenvector maps (MEMs) as a method of spatial eigenfunction. Variation partition analysis showed that 15.0% of the total bacterial community variation was explained by land-use type, 11.8% by edaphic variables, and 3.9% by spatial variables (Fig. 2D). Land-use type and edaphic variables jointly demonstrated 7.3% of the community variation, suggesting that a large proportion of variability in soil chemical properties are associated with the changes in the use of land. For different types of agricultural land use, edaphic variables (11.9% in orchard, 10.2% in paddy, and 9.8% in greenhouse soils) was shown to be higher than spatial variables (6.7% in orchard, 7.2% in paddy, and 5.9% in greenhouse soils) (Supplementary Fig. S6). Taken together, despite the various unknown factors that influence community variation, soil chemical properties derived by agricultural land use significantly affect bacterial community structures.

### Indicator taxa for specific types of agricultural land use

**Figure 3:**
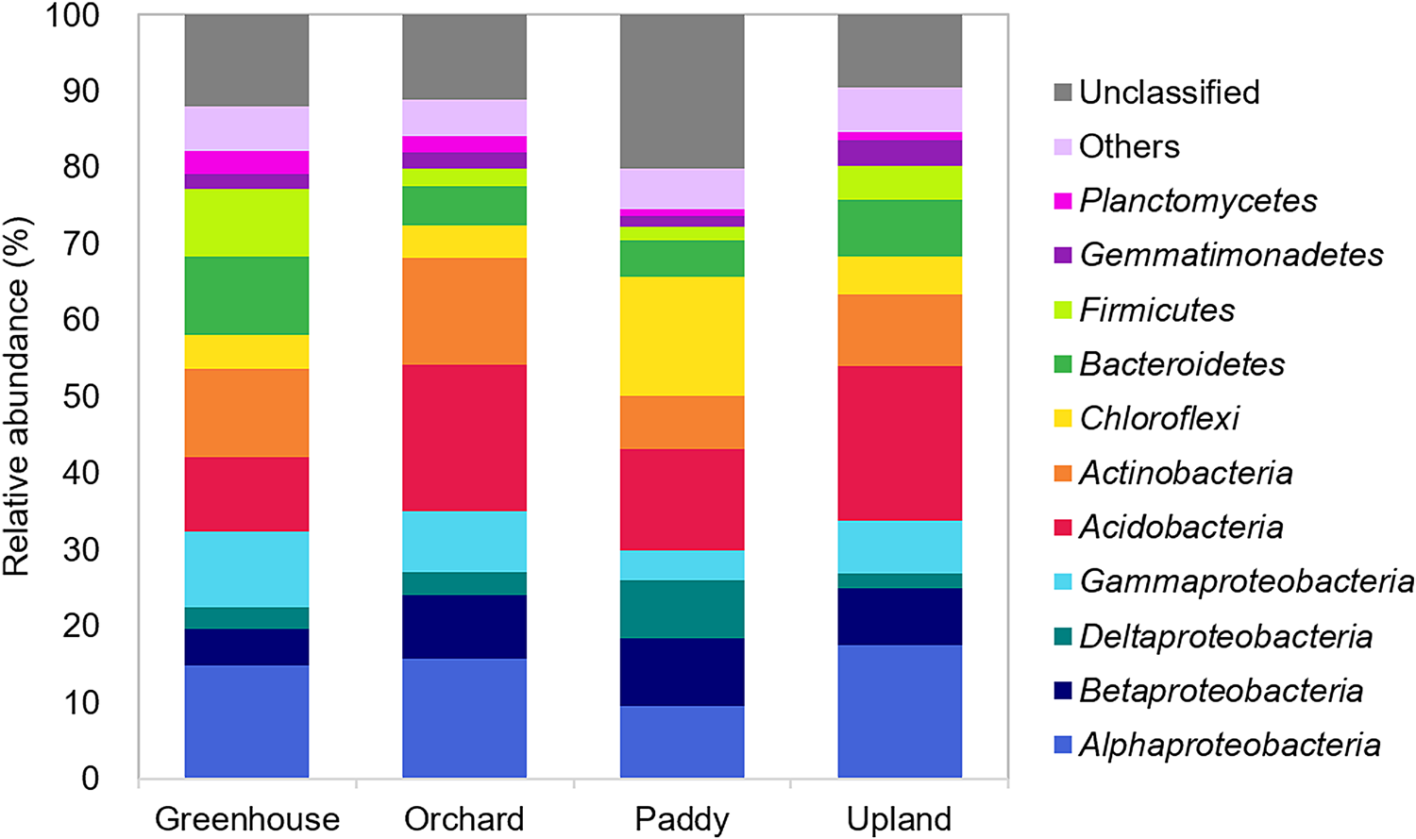
Taxonomic distribution of the bacterial communities in the four types of agricultural land use. The phyla with an abundance of < 1% are indicated as “others”. For *Proteobacteria*, the classes are indicated. The stacked column bar graph was generated using Microsoft Excel software.

Of the 68,528 OTUs obtained from 853 soil samples across four types of agricultural land, 47,095 (68.7%) OTUs were assigned to phylum-level taxa. At lower taxonomic levels, 38,572 (56.3%), 29,381 (42.9%), 20,198 (29.5%), and 12,169 (17.8%) OTUs were assigned to class, order, family, and genus, respectively. Among the 40 phyla identified in this study, eight had relative abundances greater than 1% and accounted for 81% of the total abundance, with *Proteobacteria* (32.7%), *Acidobacteria* (14.9%), and *Actinobacteria* (10.5%) being the dominant phyla of the bacterial communities across soils of different agricultural land-use types (Fig. 3). Relative abundances of *Bacteroidetes* (10.3%) and *Firmicutes* (8.8%) were higher in greenhouse soils than in other soils, while *Acidobacteria* (19.1% and 20.2%, respectively) in orchard and upland soils were more abundant than the other two soils. The relative abundances of *Chloroflexi* (15.5%) and *Deltaproteobacteria* (7.6%) in paddy soils were two to three times higher, while that of *Alphaproteobacteria* (9.5%) was lower than those of soils of the other agricultural land-use types.

**Figure 4:**
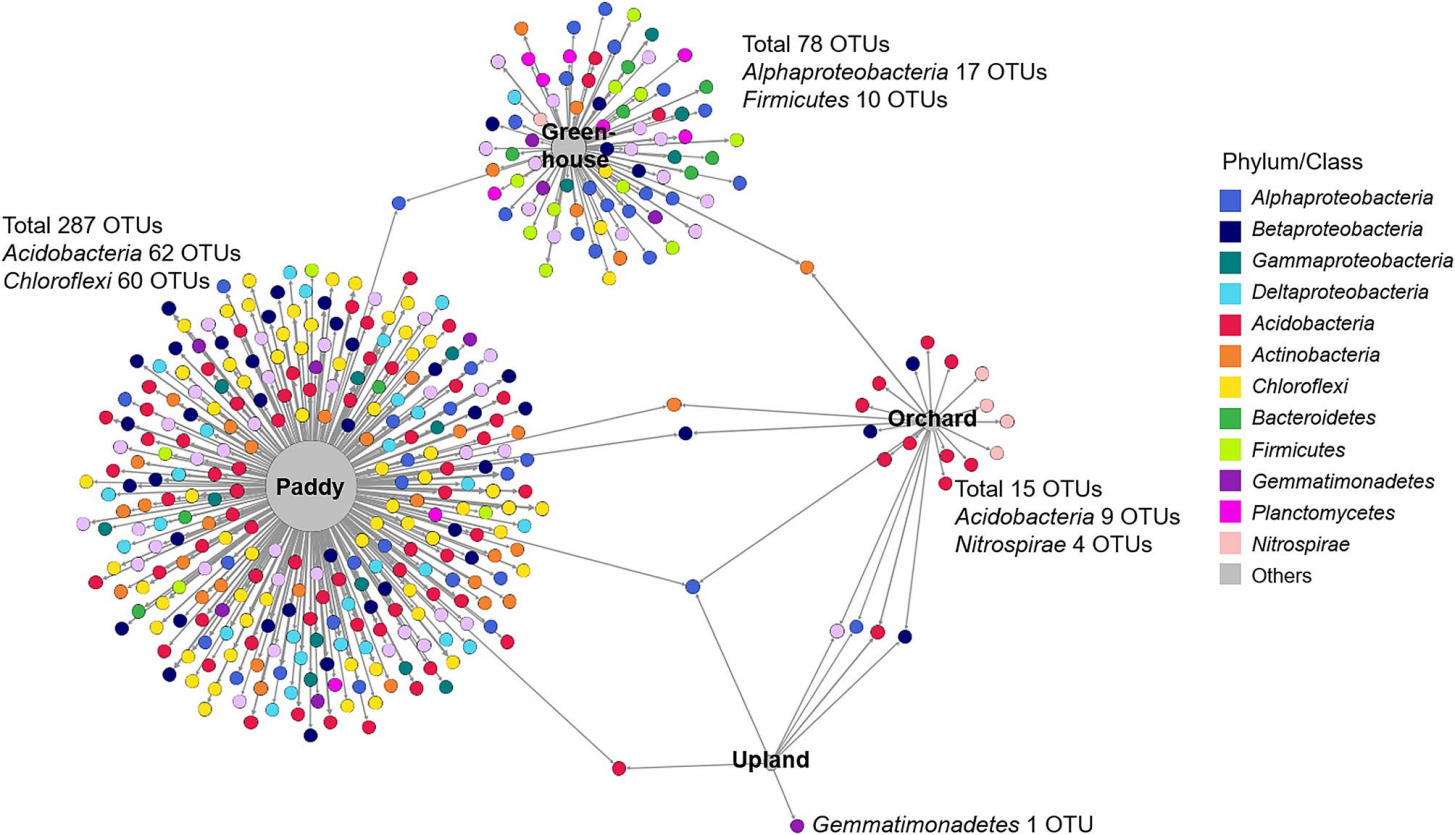
Bipartite network showing the associations between the four types of land use and 391 significantly associated OTUs (*P* < 0.01). Edges (node connection) show the association of individual OTUs with each type of agricultural land use. OTUs are colored by phylum or class. The network analysis was visualized using Gephi 0.9.1.

To identify individual OTUs sensitive to specific agricultural land-use type, indicator species analysis was performed based on point biserial correlation. The 391 OTUs had significant associations (point biserial correlation coefficient R > 0.4 and *P* < 0.001) with a particular agricultural land-use type or its combinations, which were illustrated with a bipartite network (Fig. 4). The sequence reads of these indictor OTUs accounted for 15.5% of the total number of sequences. Paddy soils had the most indicator OTUs (287), with a relative abundance of 25.6%, followed by greenhouse (78 OTUs, with a relative abundance of 10.4%), orchard (15 OTUs, with a relative abundance of 1.6%), and upland (1 OTU, with a relative abundance of 0.1%) soils, indicating that paddy soils provide a more distinctive niche than the other land-use types do. The indicator taxa of paddy soils comprised OTUs belonging mainly to the phyla *Chloroflexi* and *Acidobacteria*, and those of greenhouse soils contained OTUs belonging mainly to the phylum *Firmicutes* and the class *Alphaproteobacteria*. The orchard soils had indicator taxa belonging to *Acidobacteria*, in particular, subgroup 6 and the phylum *Nitrospirae*. The upland soils had only one specific indicator taxon, which belonged to the genus *Gemmatimonas*.

Of indicator taxa of each agricultural land-use type, the most abundant OTUs were OTU174 (0.94%), OTU7 (0.98%), OTU8 (0.45%), and OTU1562 (0.16%) in paddy, greenhouse, orchard, and upland soils, respectively (Supplementary Fig. S7). OTU174 was affiliated with *Chloroflexi* and clustered with uncultured bacterial clones detected in paddy soils. OTU7 was phylogenetically close to *Bacillus* isolated from the rhizosphere soil of cucumber and tomato, which are the main vegetables grown in greenhouses. OTU8 was affiliated with *Nitrospirae* and clustered with uncultured bacterial clones observed in soils growing trees and grasses. OTU1562 belongs to *Gemmatimonadetes* and was clustered with uncultured bacterial clones observed in cropping soils with peanut, tobacco, and vegetables. To conclude, the majority of bacterial communities in soils were not differentiated by the types of agricultural land use, and there were distinct taxa specific to agricultural land use.

### Co-occurrence networks of soil bacterial communities in different types of agricultural land use

**Figure 5:**
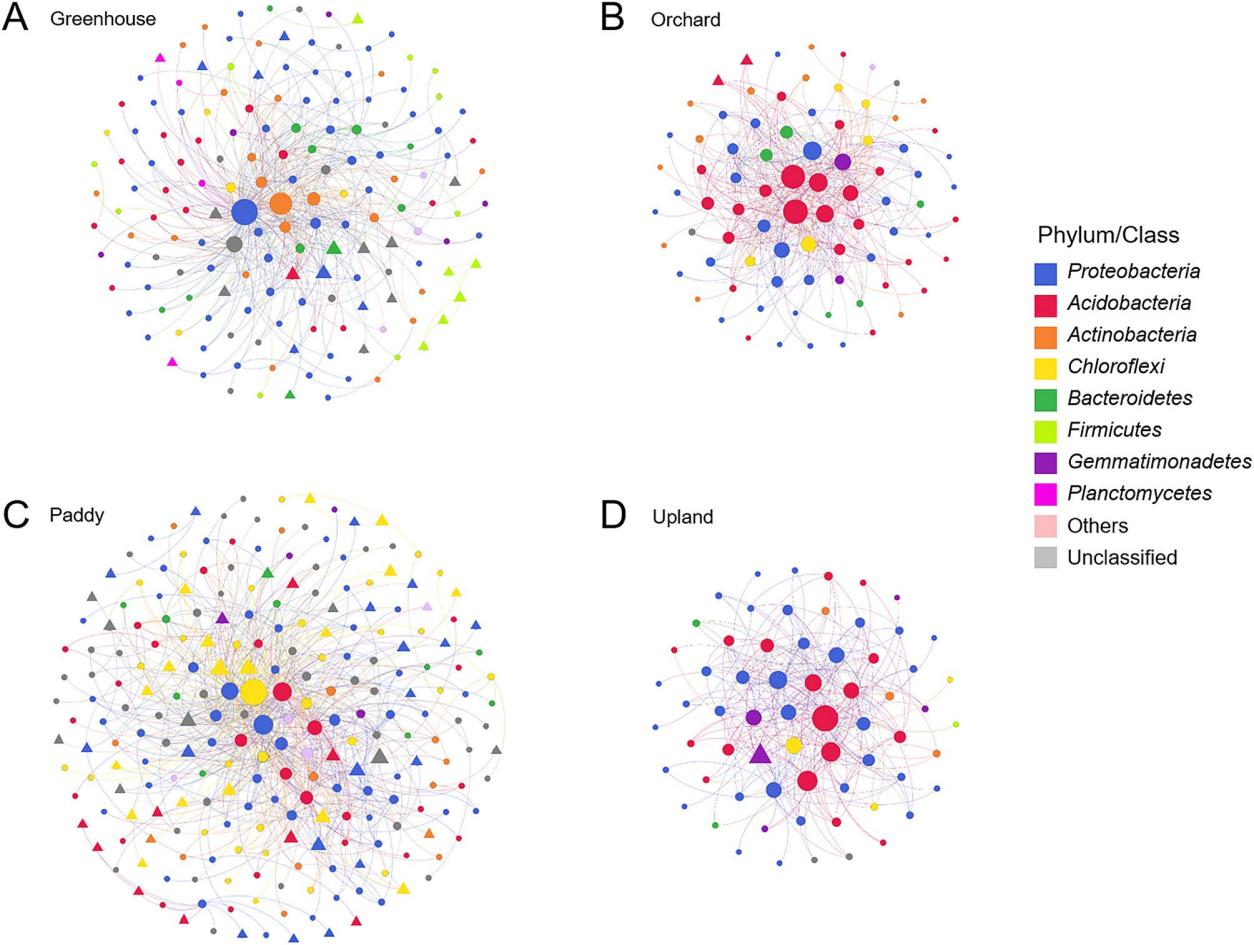
Co-occurrence networks of each type of agricultural land use. Circles and triangles indicate OTUs associated in the network. In particular, triangles represent indicator OTUs analyzed in Fig. 4. The size of circles and triangles is proportional to the number of degrees. OTUs are colored by phylum. The network analysis was visualized using Gephi 0.9.1.

To explore the complex microbial community structures in different types of agricultural land use, we performed co-occurrence network analysis using molecular ecological network analyses (MENA) based on random-metric theory (RMT). In the network analysis, common OTUs present in > 50% of samples were used. The network connectivity with a high level of R^2^ of power-law (> 0.7) indicated scale-free properties (Supplementary Table S2). The number of OTUs associated with the networks were the highest in paddy soils and the lowest in upland soils (Fig. 5). The average network distance, referred to as the average path length (GD), was the highest in the network of paddy soils. However, the connectivity between OTUs, referred to as average degree (avgK), were the highest in the network of orchard soils, followed by upland, paddy, and greenhouse soils. The results of network topology showed that relatively large numbers of bacterial taxa in the bacterial communities of paddy and greenhouse soils were associated with the co-occurrence networks but were not densely connected to each other, while relatively low numbers of OTUs associated with the networks of orchard and upland soils tended to be closely connected to each other.

Next, we identified OTUs that have the most frequent interactions with other taxa and the highest value of betweenness centrality in the co-occurrence networks, which are potential keystone taxa playing an important role in a microbial ecosystem. The keystone OTUs also varied with the types of agricultural land use: OTU46927 (phylum *Chloroflexi*) in paddy soils, OTU37000 (class *Gammaproteobacteria*) in greenhouse soils, OTU608 and OTU16 (phylum *Acidobacteria*) in orchard soils, and OTU127 (phylum *Acidobacteria*) in upland soils (Supplementary Fig. S8). None of them were identified as indicator OTUs that are highly abundant in specific types of agricultural land use, as described above. Most of the indicator OTUs in the networks showed relatively less connectivity with other OTUs (Fig. 5), indicating that keystone taxa with high connectivity are independent of indicator taxa.

## Discussion

In the present study, we investigated soil bacterial distributions across four types of agricultural land use, including greenhouses, orchards, paddy cultivation, and uplands, throughout the Republic of Korea. The bacterial diversities and community structures were clearly differentiated by agricultural land-use type (Fig. 1 and Table 1), which were represented as soil chemical properties, of which paddy soils showed the most distinct characteristics in comparison with the other land-use types; greenhouse soils also had different characteristics from those of orchard and upland soils (Fig. 2 and Table 2). The subset of bacterial taxa in the communities were specific to each land type, which were related to different phylum distributions (Figs. 3 and 4). The bacterial communities in different types of agricultural land use exhibited distinct co-occurrence patterns (Fig. 5).

### The type of agricultural land use affects soil bacterial community structures

The bacterial community structures were differentiated by the types of agricultural land use, as observed by NMDS analysis (Fig. 1). These results are consistent with findings reported by previous studies that investigated the responses of bacterial communities to agricultural management, such as conventional versus conservation agriculture practices^16,35,36^. Agricultural management practices such as irrigation, fertilization, tillage, and the application of plant protection chemicals can vary across land-use types depending on the cultivated crops. Our study aimed to conduct a nationwide survey of bacterial distribution across four different types of agricultural land use at large spatial scales; however, the impact of individual management practice on bacterial communities was not investigated. Instead, we hypothesized that the management practices in each type of agricultural land use represent soil chemical properties. Rice paddy fields are unique environments as a flooded parcel of arable land, which can be further divided into oxic surface soil and anoxic bulk soil during rice cultivation^37^. Microscale gradients of oxygen within these soil compartments influence the spatial distribution of microbial communities, leading to the highest bacterial richness and diversity (Table 1), with the most distinguishable community structure from those of the other land-use types (Fig. 1). It is generally known that bacterial richness and diversity are the highest in neutral soils and lower in acidic soils^17^. We also observed the unimodal diversity patterns in agricultural soils except for paddy soils. Most paddy soils had a rather narrow pH range (pH 5.0–6.0), which is not sufficiently broad for pattern detection. Although paddy soils had relatively lower pH values than other land-use types, their higher bacterial richness and diversity might be responsible for the distinct environments supported diverse microbial niches. Greenhouse farming is subject to intensive agricultural material inputs to enhance productivity, resulting in an accumulation of chemical components in the soil, such as available P_2_O_5_ and exchangeable cations^38^. This relates to the distinct chemical properties of greenhouse soils that differentiate it from other land-use types with significantly higher values of EC, available P_2_O_5_, and exchangeable cations (Fig. 2). These chemical factors are known to affect soil microbial community structures^31,39–43^. Although orchard and upland systems cultivated with perennial and annual plants in open fields, respectively, showed similar soil chemical properties (Table 2), crop species can affect soil microenvironments through differences in root exudates or leaf litter produced^44^, leading to different bacterial community structures in various agricultural land-use types. Overall, our results indicate that agricultural management practices corresponding to types of agricultural land use have significant impacts on soil chemical properties and drive variation in bacterial community structures.

Studies have shown that environmental variables generally change with spatial gradients, which are correlated with bacterial biogeography^23,32^. However, the soil chemical properties measured in this study did not show a spatial gradient due to intensive anthropogenic activities applied under each type of agricultural land use for crop cultivation. Variation partitioning analysis, represented by the type of agricultural land use and edaphic variables, were major determinants of bacterial community structure in agricultural soils (Fig. 2). This explains why variations in bacterial communities within each type of agricultural land use were more closely associated with soil chemical properties and that the effects of past dispersal or disturbance events were minimized.

### Distribution of bacterial taxa specific to agricultural land-use types

In this study, we aimed to identify OTUs sensitive to agricultural land-use types by performing a correlation-based indicator species analysis^45^. We identified 391 OTUs having strong and significant correlations with types of agricultural land use, indicating that specific OTUs are prevalently distributed in their preferred types of agricultural land use. These relationships further suggest ecological attributes of these taxa that are sensitive to environmental conditions of certain agricultural land-use types. In particular, paddy soils had a much greater number of indicator OTUs (287) than other types of agricultural land use. Most of the paddy indicator OTUs were related to the phylum *Chloroflexi* (Fig. 4), with a relatively higher abundance over the other three types of agricultural land use (Fig. 3). The isolates of *Chloroflexi* have been detected in anaerobic environments such as sediments, hot springs, and sludge and include mesophilic and thermophilic aerobic and anaerobic chemoheterotrophs^46–49^. *Chloroflexi* is prevalent in oligotrophic environments, such as nutrient-poor soils^50,51^ and alpine tundra soil^52^. Since flooded paddy fields provide anaerobic conditions and have lower EC, available P_2_O_5_, and exchangeable cations compared to other types of land use, the abundances of *Chloroflexi* were relatively high in rice paddy soils, consistent with previous studies^53–55^.

*Acidobacteria* was one of the abundant phyla not only in paddy but also in orchard indicator OTUs. This conflicting relationship implies that soils harbor different compositions of the phylum *Acidobacteria* at lower taxonomic levels according to agricultural land-use types: orchard-related indicator OTUs mainly comprised subgroup 6, while paddy-related indicator OTUs comprised subgroups 1, 3, 4, 7, 11, 16, and 18. Relationships between acidobacterial abundance and soil chemical properties, such as carbon amendment level and soil pH, have been reported^56,57^. In particular, Navarrete et al.^58^ described *Acidobacteria* subgroups that exhibited different correlations with soil parameters. For example, *Acidobacteria* subgroups 1, 2, and 13 had negative relationships with soil properties, such as pH, N, C, P, Ca^2+^, Mg^2+^, and K^+^, while *Acidobacteria* subgroups 4, 5, and 6 were positively correlated with these soil factors. This result is consistent with our finding wherein *Acidobacteria* subgroups had different distributions in paddy and orchard soils.

Many indicator OTUs in the greenhouse soils were associated with *Firmicutes* at a relatively higher abundance than that of the other types of agricultural land use (Fig. 4). Their high abundance in greenhouse soils, which contain relatively high levels of edaphic factors, is supported by previous studies that reported the prevalence of *Firmicutes* in copiotrophic environments, such as agricultural fields with nutrient inputs^59^ and soils with agricultural intensification^60^. In particular, OTU7 (0.98%), affiliated with *Bacillus*, was the most abundant among the greenhouse indicator OTUs, which were clustered with *Bacillus* strains isolated from the rhizosphere or endosphere of vegetables such as tomatoes and cucumbers (Supplementary Fig. S7). *Bacillus* species are beneficial bacteria well-known to promote plant growth and enhance plant tolerance to abiotic and biotic stresses^61^. Since vegetables grow continuously in greenhouse soils with fertilizers, members of *Bacillus* closely associated with these plants seem to be predominant in greenhouse soil.

We identified six OTUs that were strongly correlated with sensitivity to EC (Supplementary Fig. S4). They were assigned to *Rhodospirillaceae* (OTU_4103, OTU_5485, OTU_340, and OTU_505), *Rhodobacteraceae* (OTU_4767), and *Chloroflexi* (OTU_269). *Rhodospirillaceae* and *Rhodobacteraceae* are families in the subclass *Alphaproteobacteria* and comprise purple non-sulfur bacteria that are phototrophic in anaerobic environments^62,63^. Although EC was one of the edaphic factors higher in the greenhouse soils, only two OTUs of EC-sensitive OTUs were greenhouse-related indicator OTUs, indicating that in addition to chemical properties of soils, complex factors in different farming systems influence specific OTUs. Our results highlight the potential of these OTUs as applicable biological indicators for monitoring how soil conditions are affected by agricultural managements.

### Effects of agricultural land-use type on co-occurrence networks

We explored bacterial co-occurrence patterns in different types of agricultural land use with a large collection of soil samples from across the Republic of Korea. The microbial interactions in networks show the structure and dynamics of soil microbial communities^27^. The members associated with the network and its topologies clearly varied with the types of agricultural land use (Fig. 5, Supplementary Table S2). Consistent with higher species richness in paddy soils, a relatively large number of OTUs were associated with the network of paddy soils. Moreover, the number of modules was the highest in the microbial network of paddy soils. Given that a module is a cluster of densely interconnected nodes and indicates groups of taxa with overlapping niches^28^, it seemed to reflect multiple niches caused by the unique environmental feature of paddy soil. In contrast, microbial networks of orchard soils were relatively small, but the interactions were close to each other. In an analysis of microbial co-occurrence patterns in forest, grassland, crop system, and vineyard soils, the network complexity was found to be lowered by high cropping intensity^64^. As orchards undergo less tillage and fruit trees are continuously grown for several years, the cropping intensity of orchards is relatively lower than that of other agricultural land-use types such as greenhouse and paddy cultivation. This might explain the more complex microbial interactions in orchard soils.

The highly connected OTUs, referred to as keystone taxa, also varied with the types of agricultural land use (Fig. 5 and Fig S3). The keystone taxa in the networks of orchard and upland soils were members of *Acidobacteria*, which is the phylum enriched in both soils. Given the similar network topology and beta-diversity of bacterial communities in orchard and upland soils, these two agricultural land-use types might have similar agro-ecosystems, which can be inferred from similar chemical properties between the two types. The keystone taxa in the microbial networks of greenhouse and paddy soils were affiliated with *Gammaproteobacteria* and *Chloroflexi*, respectively, which were also the relatively more abundant phyla in these soils compared to other soils studied. Although keystone taxa are known to play important roles in microbial communities, the relative abundances of the keystone OTUs were low, and none of them were indicator taxa. Most indicator taxa associated with the microbial networks had a few links. This result is consistent with the study that most indicator OTUs in the soil microbiome with different cropping practices, including organic managements and tillage intensities, were not keystone taxa^65^, suggesting that keystone taxa were not significantly affected by environmental disturbances, but those indicator taxa were affected by agricultural activities.

## Conclusion

The soil environment is a dynamic and highly complex system composed of microbes that are affected by various biotic (i.e., earthworms, arthropods, and microbial domains) and abiotic (i.e., precipitation, temperature, humidity, and anthropogenic effects) factors. Our study showed that agricultural land-use types determined bacterial community structures and specific taxa were enriched in specific types of agricultural land use, with distinct correlations with soil chemical properties. Furthermore, microbial interactions based on the co-occurrence patterns in soil bacterial communities also varied with agricultural land-use types. Our findings provide a novel perspective of how land-use type-specific taxa reflect soil conditions and can thus be used as potential biological indicators for maintaining soil health and sustainable crop production. Further research is needed to explore relationships between soil fertility, crop productivity, and microbial community structure, which will help us better understand which bacterial communities or specific taxa support sustainable agricultural management.

## Materials and methods

### Soil sampling

Soil sampling was conducted between March 2013 and May 2016 throughout the Republic of Korea (Supplementary Fig. S1 and Supplementary Data S1). Soil samples were collected from the southern regions in March and the northern regions in May to decrease temperature variation, except for the paddy fields, where samples were taken from March to April, before the flood period. A total of 853 soil samples were collected and grouped into four types of agricultural land use, namely, greenhouse, orchard, rice paddy, and upland. Sampling was conducted in the order of uplands (2013), orchards (2014), paddy fields (2015), and greenhouses (2016). At each sampling site, a total of ten soil cores were taken at 10 m intervals to a depth of 15 cm, pooled together in a sterile plastic bag, and transported to the laboratory in an ice-filled cooler. The latitude and longitude of each sampling site were recorded using a hand-held global positioning system.

### Determination of soil physicochemical properties

Soil samples were mixed well and sieved through a 2 mm mesh. Samples were pre-incubated at 22 °C for 7 days to reduce environmental disturbances during sampling and sieving^66^ and stored at − 80 °C until further molecular analysis. Soil pH and electrical conductivity (EC) were measured using a pH meter (CyberScan pH1500; EUTECH, USA) and an EC meter (D-54; Horiba, Japan), respectively, after shaking the soil/water (1:5) mixture for 30 min at 200 rpm. The organic matter (OM) content was measured using the Walkely and Black method^67^, and the available P_2_O_5_ content was measured by the Lancaster method^68^. Exchangeable cations (Ca^2+^, Mg^2+^, Na^+^, and K^+^) were extracted with 1 M NH_4_OAc (pH 7.0) from soil samples and analyzed using inductively coupled plasma atomic emission spectroscopy (ICP-AES; GBC Integra-XMP, Melbourne, Australia).

### DNA extraction and 16S rRNA pyrosequencing

DNA was extracted from approximately 0.5 g of the soil samples in duplicate using the FastDNA SPIN Kit for Soil (MP Biomedicals, Solon, OH, USA) according to the manufacturer’s instructions. The DNA extracts were quantified using an ND-1000 spectrophotometer (NanoDrop Technologies, Wilmington, DE, USA). Integrity of DNA was confirmed by running the DNA extracts on a 1.2% (w/v) agarose gel with 0.5X TBE buffer (45 mM Tris–borate, 1 mM EDTA, pH 8.0).

PCR amplification, purification, and pyrosequencing of partial 16S rRNA genes were performed at the National Instrumentation Center of Environmental Management (NICEM; Seoul, Republic of Korea) using the 454 GS FLX Titanium Sequencing System (Roche 454 Life Sciences, Branford, CT, USA). Briefly, PCR amplification was performed using the specifically designed fusion primers V1-9F (5ʹ-X-AC-GAGTTTGATCMTGGCTCAG-3ʹ) and V3-541R (5ʹ-X-AC-WTTACCGCGGCTGCTGG-3ʹ), which contained linker sequences (AC) and 7–10 barcode sequences, under the following conditions: initial denaturation at 94 °C for 5 min, followed by 10 cycles of 94 °C for 30 s for denaturation, 60 °C for 45 s for annealing, and 72 °C for 90 s for elongation, with the annealing temperature reduced by 0.5 °C per cycle from the preceding cycle. Twenty additional cycles of 94 °C for 30 s, 55 °C for 45 s, and 72 °C for 90 s were performed ^31^. Each PCR mixture (50 μL) included 5 μL of 10X buffer, 1 μL of dNTP mix (10 mM), 1 μL of each fusion primer (50 pmol), 40.8 μL of sterile deionized H_2_O, 1 μL of *Taq* DNA polymerase (1 U), and 1 μL of template DNA (1 ng). PCR products were pooled at equimolar concentrations for pyrosequencing reactions.

### Analysis of pyrosequencing data

Clustering of 16S rRNA amplicon sequence reads into operational taxonomic units (OTUs) was performed using the UPARSE pipeline^69^ with some modifications. Briefly, barcode and primer sequences were removed using the trim.fastq script. Reads shorter than 300 bp were removed, and those longer than 300 bp were properly trimmed. Reads were then clustered into OTUs at a cutoff of 0.03 using the UPARSE-OTU algorithm. Chimeras were removed in de novo and reference modes using UCHIME and USEARCH, respectively. The resulting OTU table was transformed using customized perl scripts for use in the MOTHUR program^70^. Taxonomic assignment was carried out using the classify.seqs command (iters = 1,000 and cutoff = 60) in the MOTHUR program based on the Ribosomal Database Project database (RDP version 14; https://rdp.cme.msu.edu). After the read number in each sample was normalized to that of the sample with the smallest number of reads (1,002 reads), Good’s coverage, richness estimator (abundance-based coverage estimator (ACE) and Chao1), and diversity indices (Shannon and inverse-Simpson) were calculated using the MOTHUR program.

### Bioinformatics analyses

Statistical analyses in this study were performed using the R program ver. 3.3.1; R Core Team^71^. Variations in bacterial community structures among the different land-use types were visualized using non-metric multidimensional scaling (NMDS) based on the Bray–Curtis distance of Hellinger-transformed OTU numbers using *metaMDS* and *decostand* functions in the *vegan* package. The dispersion of bacterial communities was measured using the *betadisper* function in the *vegan* package. Analysis of similarities (ANOSIM) and permutational multivariate analysis of variance (PERMANOVA) were performed for pair-wise comparisons of soil microbial communities with four different types of agricultural land use using the *anosim* and *adonis* functions in the R package vegan, respectively. Soil chemical parameters were log-transformed for normal distribution, while raw data was used for pH, and variations were visualized using principal component analysis (PCA). The constrained ordination analysis of bacterial communities by soil chemical properties was performed using redundancy analysis (RDA) with the *rda* function in the R package. The correlation between soil chemical properties and relative abundances of OTUs were examined using the *cor.test* function based on Pearson’s correlation method. The statistical analysis of distance-decay patterns, and the relationship between bacterial diversity and soil pH were performed using quadratic regression. Variation partitioning analysis was performed using the *varpart* function in the *vegan* R package to assess the relative influence of edaphic variables, land-use type, and geographic distance on bacterial community structures. To perform spatially explicit multiscale modeling, spatial variables were calculated using the MEMs approach in *adespatial* R package^72^. The connectivity matrix (relative neighborhood) weighted by the Euclidian distance function was generated, followed by a forward selection procedure that was used to select the set of MEM variables which best explained the community variation based on adjusted the *R*^2^ statistics.

To identify the OTUs that were specifically abundant in each type of agricultural land use, indicator species analysis was conducted using the *multipatt* function with the *r.g* option in the R package *indispecies*. The strength of association of each OTU with a particular agricultural land-use type or its combinations was represented as a point-biserial correlation coefficient *R* using correlation-based indicator species analysis^45^. The false discovery rate (FDR) was used for multiple comparison correction^73^ using the R package FSA^74^. The bipartite network was visualized with Gephi 0.9.1^75^.

Co-occurrence network analysis was conducted using molecular ecological network analyses (MENA) based on random-metric theory (RMT)^76^. To reduce environmental disturbance, datasets were separated by the types of agricultural land use, and OTUs detected in > 50% of samples were used in the analysis. The detailed options of MENA were as follows: 0.01 was filled in the blanks with paired valid values; logarithm values were obtained; Pearson’s correlation coefficient was used for correlation analysis; and calculations were done by decreasing the cutoff from the top with Poisson regression only. A similarity threshold was selected automatically by the RMT-based approach to define the adjacency matrix. The results of the network analysis were visualized using Gephi 0.9.1^75^.

## Supplementary information

Supplementary file1

Supplementary file2

## Data Availability

Raw pyrosequencing data obtained in this study were deposited in the NCBI Sequence Read Archive (https://www.ncbi.nlm.nih.gov/sra) with the accession number PRJNA499124.
