# Supplementary material for: Different types of agricultural land use drive distinct soil bacterial communities
Source: Sci Rep. 2020 Oct 15;10:17418. doi: 10.1038/s41598-020-74193-8 (PMC7562711; doi:10.1038/s41598-020-74193-8)
Supplement: Supplementary file 1 — Supplementary file1 [file 41598_2020_74193_MOESM1_ESM.docx]

**Supplementary Information**

**Different types of agricultural land use drive distinct soil bacterial communities**

**Authors:** Shin Ae Lee^1,6^, Jeong Myeong Kim^2, 6^, Yiseul Kim^1^, Jae-Ho Joa^3^, Seong-Soo Kang^4^, Jae-Hyung Ahn^1^, Mincheol Kim^5^, Jaekyeong Song^1^, Hang-Yeon Weon^1*^

**Affiliations:**

^1^Agricultural Microbiology Division, National Institute of Agricultural Sciences, Rural Development Administration, Wanju, Republic of Korea

^2^Water Supply and Sewerage Research Division, National Institute of Environmental Research, Incheon, Republic of Korea

^3^Research Institute of Climate Change and Agriculture, National Institute of Horticultural and Herbal Science, Rural Development Administration, Jeju, Republic of Korea

^4^Soil and Fertilization Division, National Institute of Agricultural Sciences, Rural Development Administration, Wanju, Republic of Korea

^5^Arctic Research Center, Korea Polar Research Institute, Incheon, Republic of Korea

^6^These authors contributed equally to the work.

**^*^Correspondign Author:** Hang-Yeon Weon (why@korea.kr)

**Supplementary Table S1.** Pair-wise comparison between the types of agricultural land use based on bacterial communities.

|  | ANOSIM | | PERMANOVA | |
| --- | --- | --- | --- | --- |
|  | R | *P* | R^2^ | *P* |
| Greenhouse vs. orchard | 0.5112 | <0.001 | 0.0834 | <0.01 |
| Greenhouse vs. paddy | 0.9381 | <0.001 | 0.1595 | <0.01 |
| Greenhouse vs. upland | 0.4754 | <0.001 | 0.0593 | <0.01 |
| Orchard vs. paddy | 0.8784 | <0.001 | 0.1827 | <0.01 |
| Orchard vs. upland | 0.2068 | <0.001 | 0.0273 | <0.01 |
| Paddy vs. upland | 0.8408 | <0.001 | 0.1314 | <0.01 |

ANOSIM, analysis of similarities; PERMANOVA, permutational multivariate analysis of variance.

**Supplementary Table S2.** Topological properties of the networks of bacterial communities in four different types of agricultural land use.

| Network indices | Greenhouse | Orchard | Paddy | Upland |
| --- | --- | --- | --- | --- |
| No. of commonly present OTUs (>50%) | 392 | 369 | 627 | 182 |
| No. of nodes | 162 | 83 | 236 | 65 |
| No. of links | 414 | 333 | 675 | 255 |
| R^2^ of power-law | 0.872 | 0.861 | 0.821 | 0.787 |
| Average degree (avgK) | 5.111 | 8.024 | 5.72 | 7.846 |
| Average path distance (GD) | 2.878 | 2.459 | 3.148 | 2.389 |
| No. of modules | 8 | 6 | 10 | 3 |


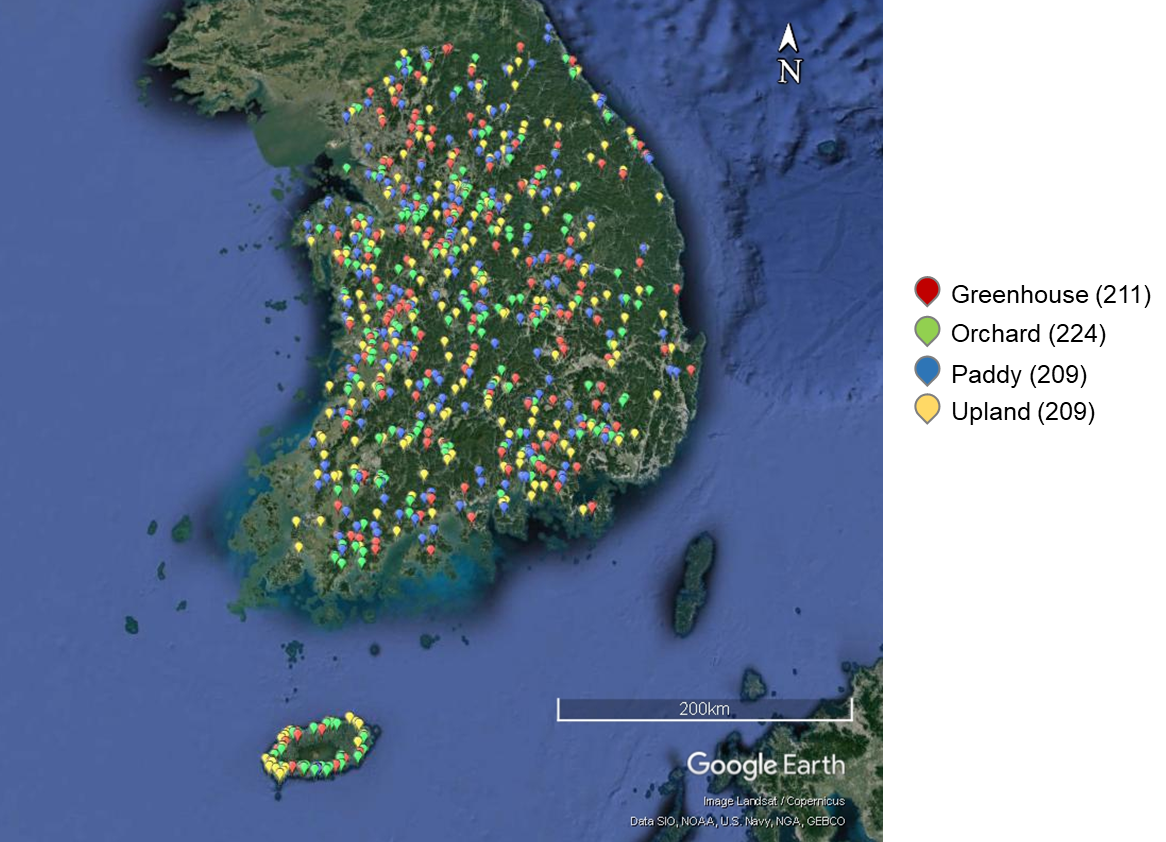


**Supplementary Figure S1.** Geographical locations of 853 sampling sites. Color markers represent the coordinates (longitude and latitude) of sampling sites on the map using Google Earth Pro 7.3.3.

**
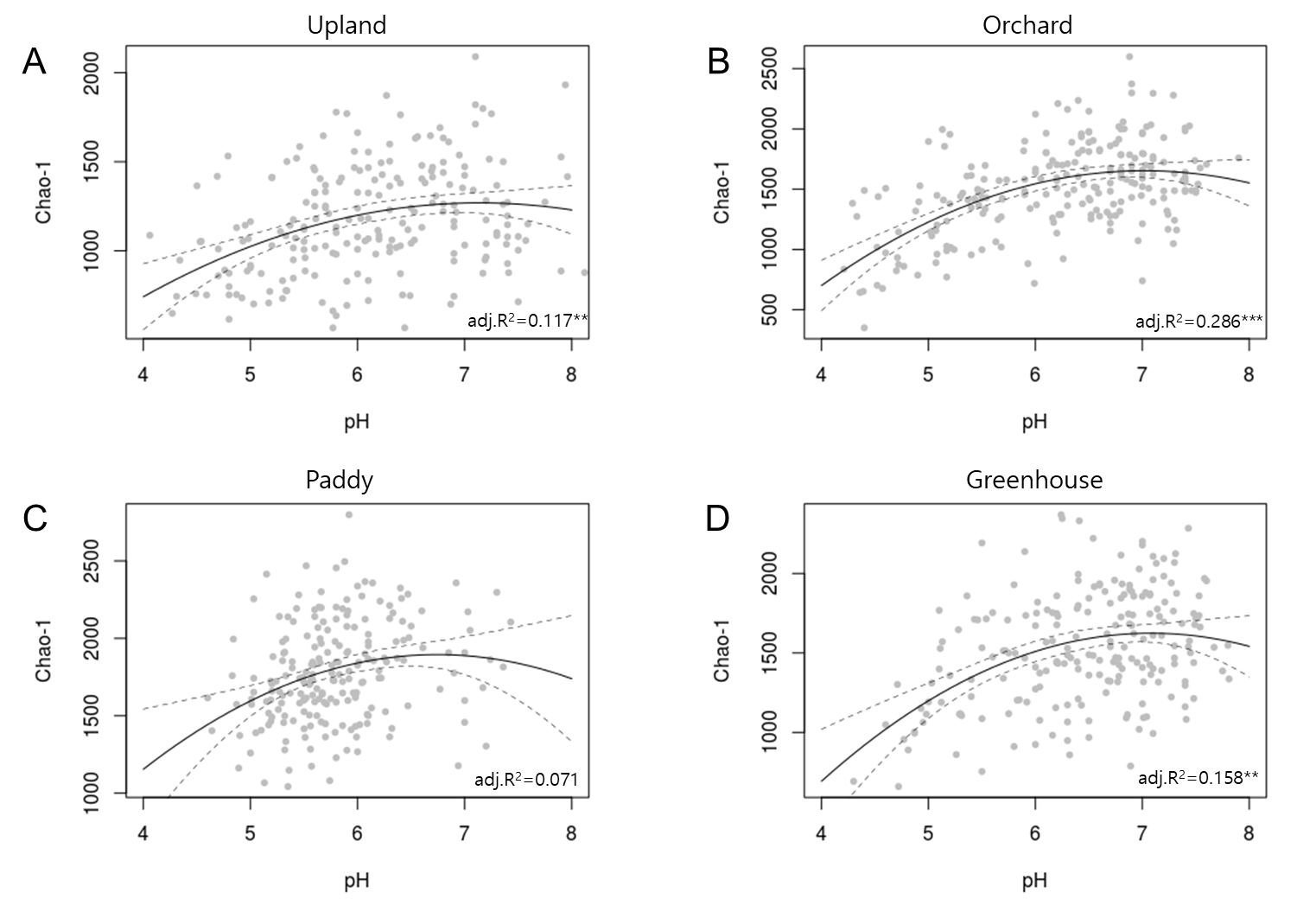
**

**Supplementary Fig. S2.** The relationship between bacterial richness and soil pH in different types of agricultural land use including upland (A), orchard (B), paddy (C), and greenhouse (D). Bacterial richness was estimated by chao-1. Quadratic regression was used to determine adjusted R^2^ values and statistical significance (****P* < 0.001 and ***P* < 0.01).

**
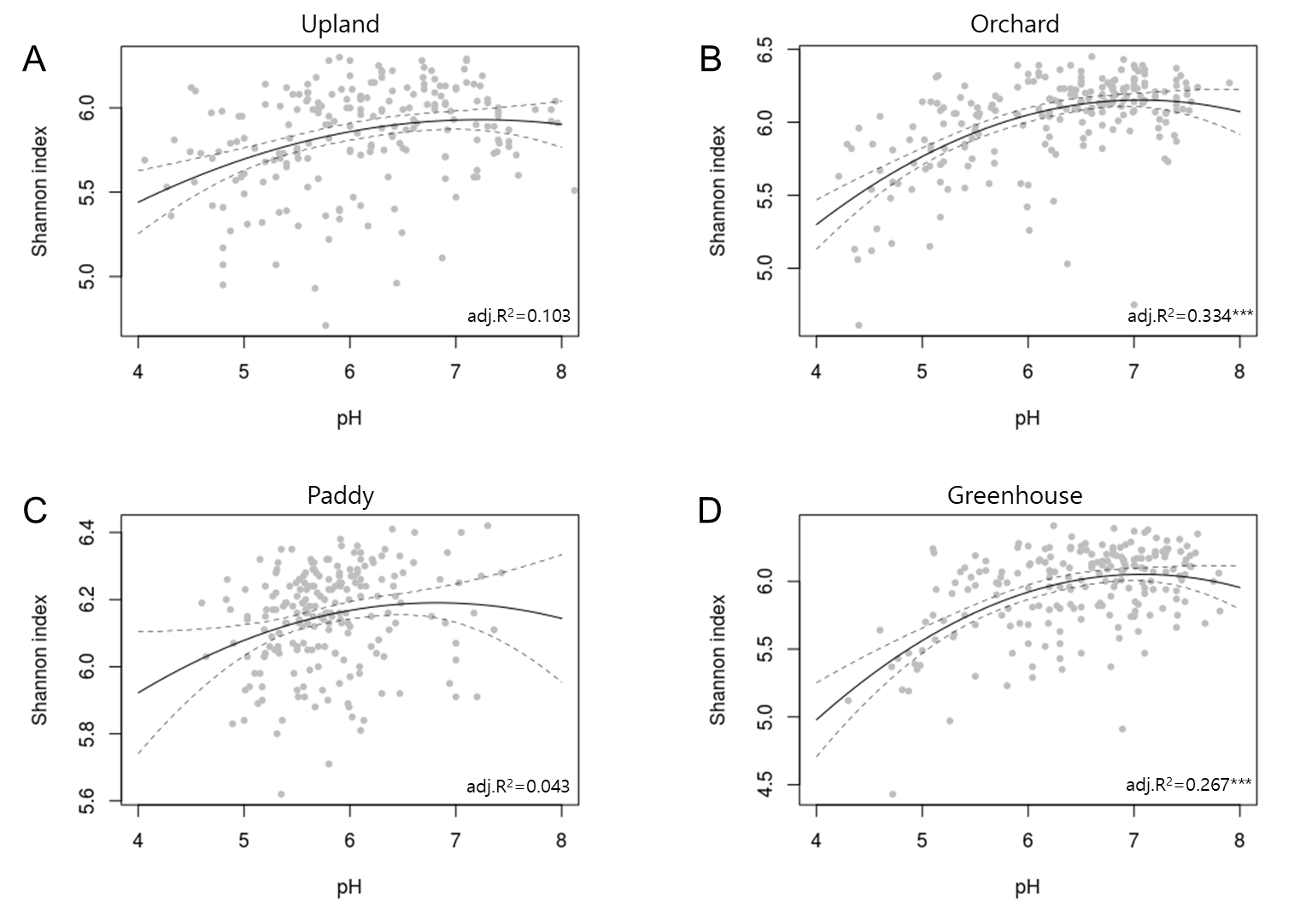
**

**Supplementary Fig. S3.** The relationship between bacterial diversity and soil pH in different types of agricultural land use including upland (A), orchard (B), paddy (C), and greenhouse (D). Bacterial diversity was estimated by Shannon index. Quadratic regression was used to determine adjusted R^2^ values and statistical significance (****P* < 0.001).


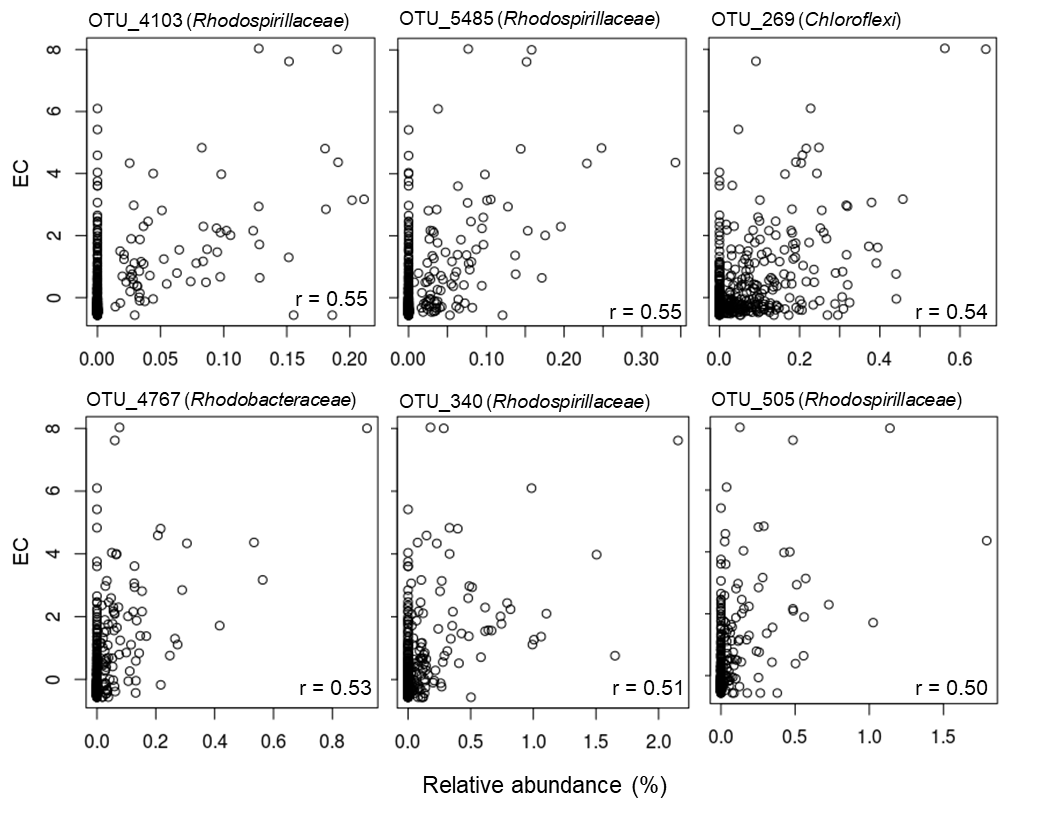


**Supplementary Figure S4**. The OTUs correlated with EC values based on the Pearson correlation method. The OTUs with Pearson’s correlation coefficient (r) > 0.5 are shown.


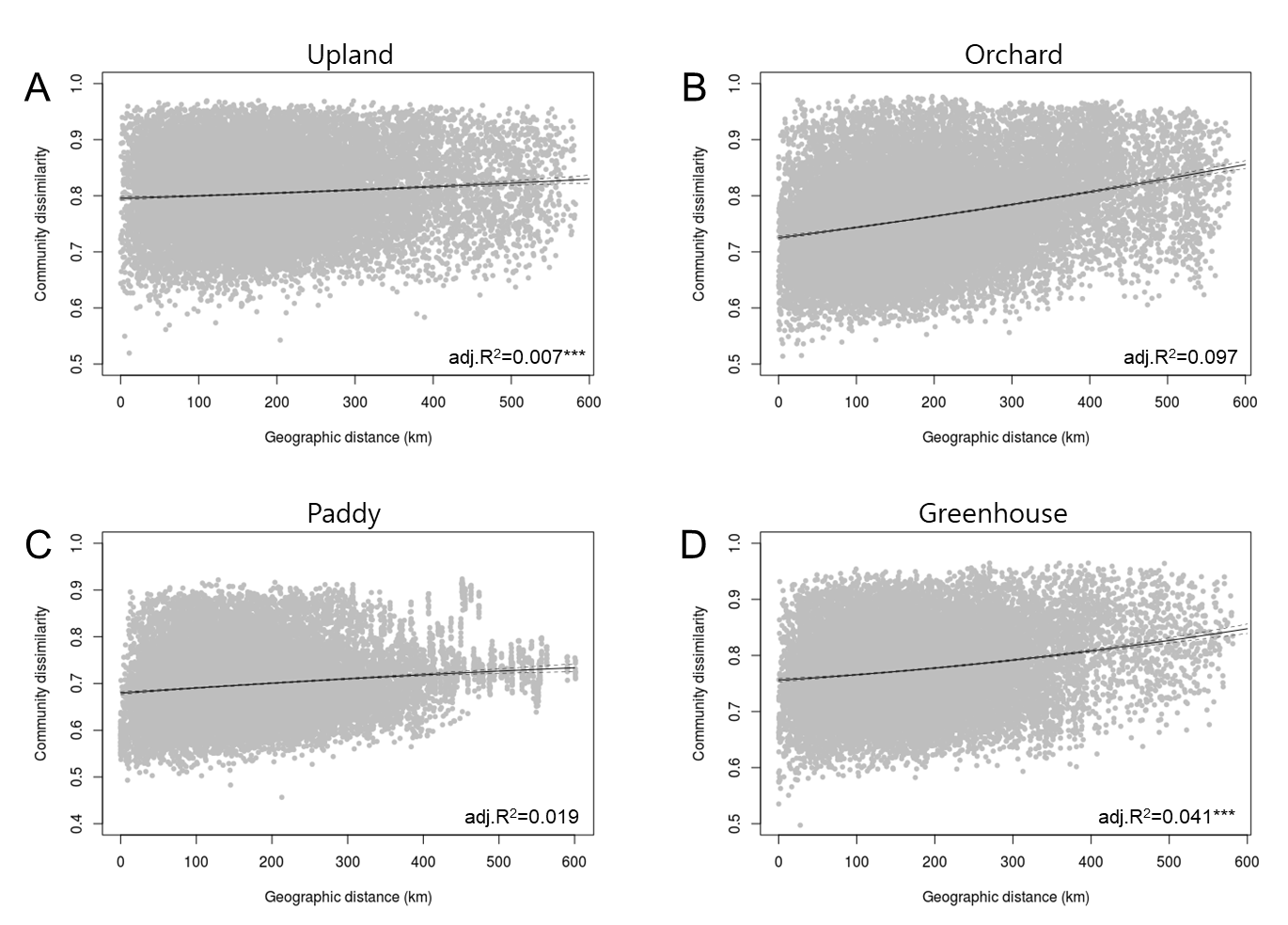


**Supplementary Figure S5.** Distance-decay biogeographic patterns of bacterial communities in different types of agricultural land use including upland (A), orchard (B), paddy (C), and greenhouse (D). Bacterial community dissimilarity was calculated using the Bray–Curtis method. Quadratic regression was used to determine adjusted R^2^ values and statistical significances (****P* < 0.001).

**
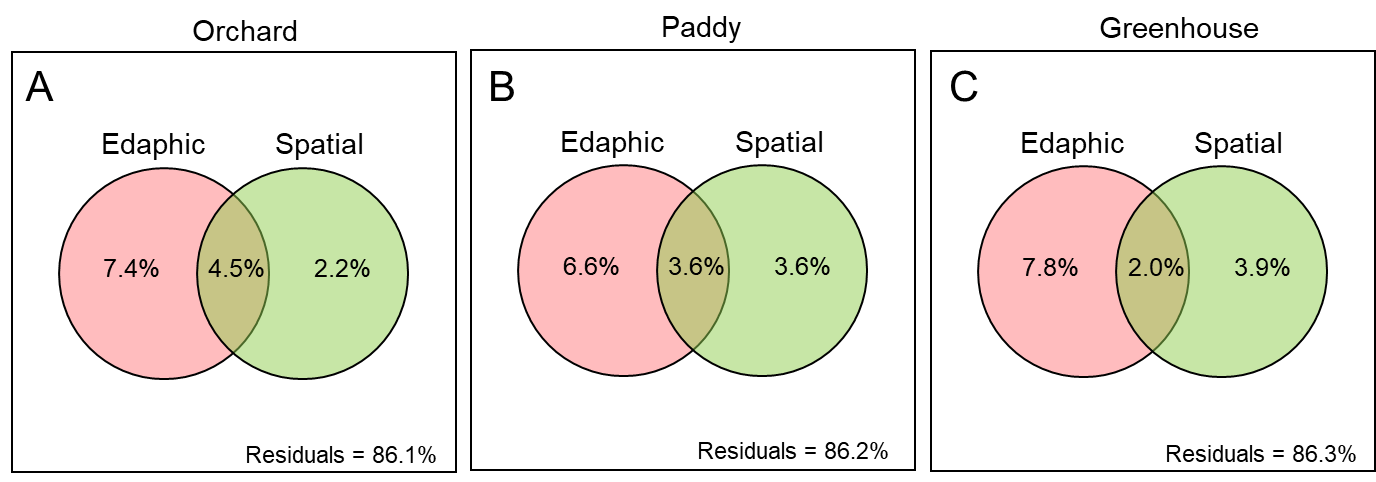
**

**Supplementary Figure S6.** Variation partitioning of bacterial communities in orchard (A), paddy (B), and greenhouse (C) soils. Venn diagram showing variation partitioning results explained by edaphic and spatial variables. There were no significant spatial variables related to upland soil communities.


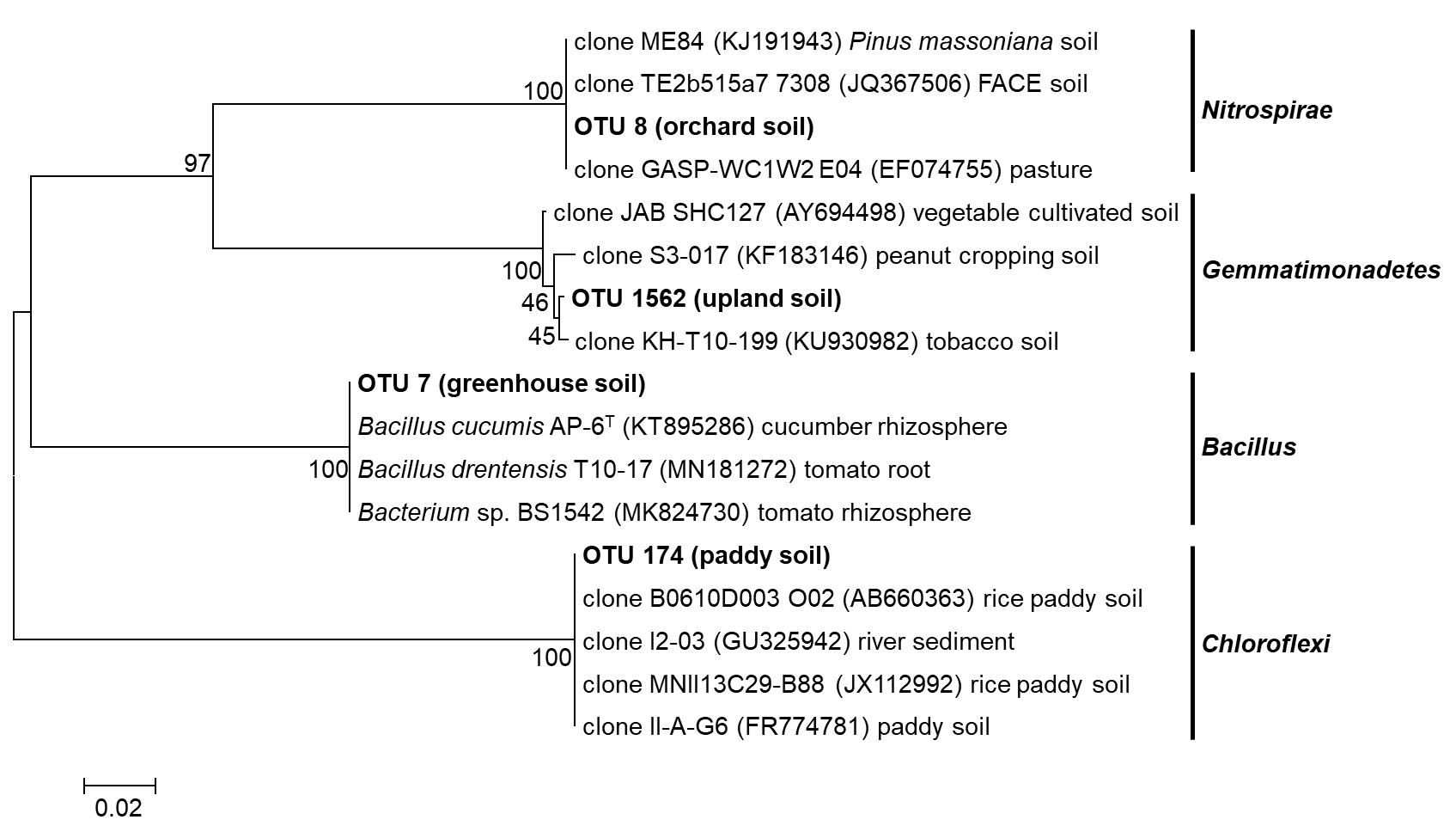


**Supplementary Figure S7.** Phylogenetic positions of the indicator OTUs in different agricultural land-use types. The phylogenetic tree is constructed based on the neighbor-joining method with representative OTU sequences and reference sequences with high sequence similarity with OTUs. The numbers next to tree nodes indicate percentage bootstrap values from 1,000 iterations. The scale bar indicates 0.02 estimated changes per nucleotide. The sources of isolates and clones are indicated for reference sequences.


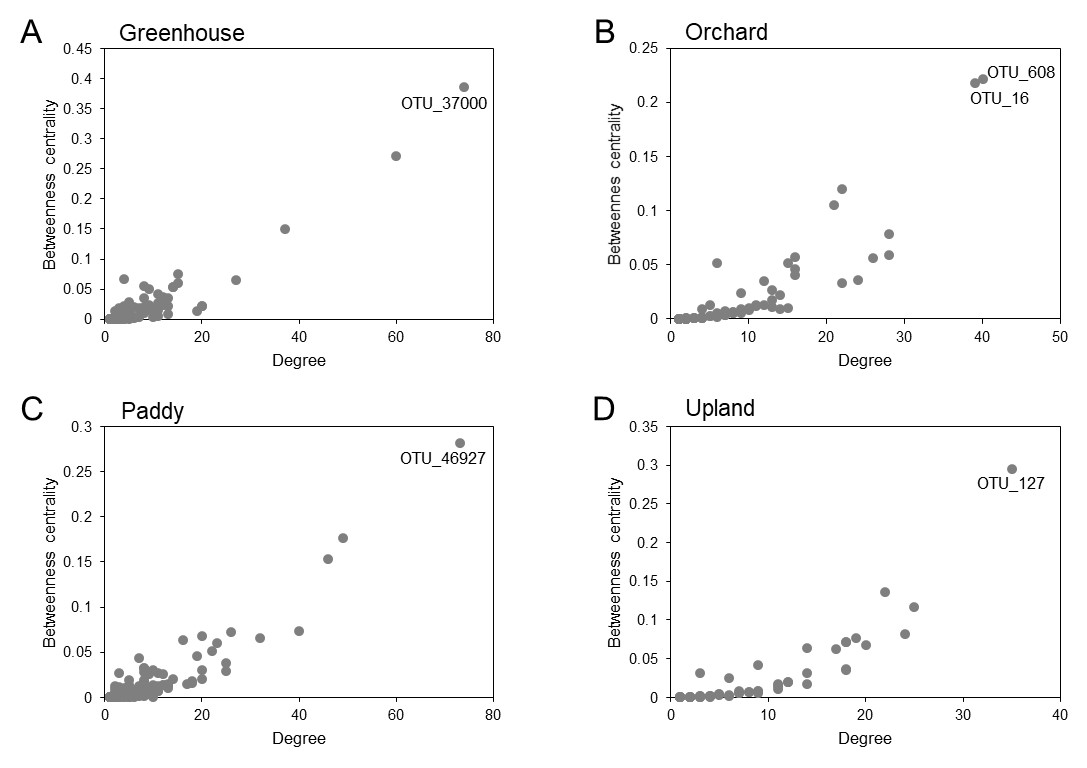


**Supplementary Figure S8.** Keystone taxa in the co-occurrence networks of greenhouse (A), orchard (B), paddy (C), and upland (D) soils. The OTUs with highest degree and betweenness centrality indicate potential keystone taxa in the complex microbial interactions.
